# The Proper Climate for Cancerous and Consumptive Cases

**Published:** 1872-06-15

**Authors:** Edward Andrews

**Affiliations:** Prof. of Surgery in Chicago Med. College


### Original Communications.

### THE PROPER CLIMATE FOR CANCEROUS AND CONSUMPTIVE CASES.

BY EDWARD ANDREWS,

*Prof. of Surgery in Chicago Med. College.*

Some years ago, I called the attention of the profession for the first time to the fact, that there is a distinct climatic cause of cancer, and that it prevails most in precisely the same regions with consumption. I based this conclusion on calculations from the figures in the volumes of Mortality Statistics of the U. S. Census of 1860.

Having recently received advance sheets of the forthcoming Census Report of 1870 I have repeated this investigation with full confirmation of the same discovery. In brief, the law of latitude and of proximity to the sea, long known as affecting the prevalence of consumption, and first clearly enunciated, I believe, by the Surgeon General of the U. S. Army, applies in full force to cancer, as the subjoined figures will show. The law may be stated as follows:

1. Consumption and Cancer prevail most near the sea, and diminish as you recede from it.

2. At equal distance from the sea they prevail most at the north and diminish as you go south.

Thus if you begin at Massachusetts and go westward, the proportion of deaths from consumption to deaths from all causes diminishes regularly as you recede from the Atlantic. Here are the figures:

*Deaths from Consumption.*

| | |
|---|---|
| Massachusetts, | 25 per cent. |
| New York, | 20 " " |
| Ohio, | 16 " " |
| Indiana, | 14 " " |
| Illinois, | 11 " " |
| Missouri, | 9 " " |
| Kansas, | 8 " " |
| Colorado, | 8 " " |
| Utah, | 6 " " |

Then if you go down to California it increases to 14 per cent in consequence of the proximity of the Pacific Ocean.

The same decrease is observed if you go from north to south, as follows:

| | |
|---|---|
| Minnesota, | 14 per cent. |
| Iowa, | 12 " " |
| Missouri, | 9 " " |
| Arkansas, | 5 " " |

Then if you go on to Louisiana it increases to 8 per cent. in consequence of the proximity of the Gulf of Mexico, aided perhaps by the marshes of the delta of the Mississippi.

It follows that the best resort for a patient threatened with cancer or consumption, is one which is at the same time as far from the sea, and as far South as possible. Such a place is New Mexico, where the deaths from consumption are only 3 per cent., or Arkansas where they are 5 per cent. Probably the central table lands of Old Mexico will be found still better when the progress of civilization shall afford safety there to travelers. Entirely in accordance with this rule, though contrary to the popular notion, we find that Minnesota is a worse place than Illinois, having 14 per cent. of deaths from consumption while our own State has only 11 per cent. The best places in the United States are

| | |
|---|---|
| New Mexico, | 3 per cent. |
| Arkansas, | 5 " " |
| Texas, | 5 " " |
| Georgia, | 5 " " |
| South Carolina, | 5 " " |
| Florida | 6 " " |
| Alabama, | 6 " " |
| Mississippi, | 6 " " |
| Utah, | 6 " " |

A marked feature of the new census is that consumption is considerably increased as compared with 1860, in nearly all the southern states and territories, while it is diminished or stationary in most of the northern states. This indicates probably an increased influx of invalids from the north to the south in search of health. In future it is to be hoped that the census will distinguish between cases native to the region, and those immigrating in pursuit of health.

By consulting the following table, the physician can see at a glance the best resorts for patients consulting him for cancer and consumption.

RATIO OF DEATHS FROM CANCER AND CONSUMPTION TO DEATHS FROM ALL CAUSES IN THE VARIOUS UNITED STATES AND TERRITORIES, FOR THE YEARS 1860 AND 1870.

| STATES AND TERRITORIES. | 1860. Cancer. | 1860. Consumption. | 1870. Cancer. | 1870. Consumption. | Average of the two Censuses. Cancer. | Average of the two Censuses. Consumption | Per Cent. of Consumption |
|---|---|---|---|---|---|---|---|
| Alabama, | 1 death to 174 | 1 death to 20 | 1 death to 124 | 1 death to 14 | 1 to 149 | 1 to 17 | 6 pr. ct. |
| Arkansas, | 1 " 277 | 1 " 27 | 1 " 204 | 1 " 14 | 1 to 240 | 1 to 20 | 5 " |
| California, | 1 " 180 | 1 " 7 | 1 " 104 | 1 " 7 | 1 to 142 | 1 to 7 | 14 " |
| Colorado, | | | 1 " 125 | 1 " 12 | 1 to 125 | 1 to 12 | 8 " |
| Connecticut, | 1 " 80 | 1 " 5 | 1 " 43 | 1 " 6 | 1 to 61 | 1 to 5 | 20 " |
| Dakota, | | | 1 " 101 | 1 " 8 | 1 to 101 | 1 to 8 | 12 " |
| Delaware, | 1 " 108 | 1 " 6 | 1 " 96 | 1 " 5 | 1 to 102 | 1 to 5 | 20 " |
| Dist. of Columbia, | 1 " 116 | 1 " 5 | 1 " 168 | 1 " 5 | 1 to 142 | 1 to 5 | 20 " |
| Florida, | 1 " 148 | 1 " 16 | 1 " 141 | 1 " 17 | 1 to 144 | 1 to 16 | 6 " |
| Georgia, | 1 " 158 | 1 " 26 | 1 " 104 | 1 " 15 | 1 to 131 | 1 to 20 | 5 " |
| Illinois, | 1 " 157 | 1 " 9 | 1 " 118 | 1 " 9 | 1 to 137 | 1 to 9 | 11 " |
| Indiana, | 1 " 176 | 1 " 9 | 1 " 105 | 1 " 6 | 1 to 140 | 1 to 7 | 14 " |
| Iowa, | 1 " 129 | 1 " 9 | 1 " 110 | 1 " 7 | 1 to 119 | 1 to 8 | 12 " |
| Kansas, | 1 " 174 | 1 " 13 | 1 " 207 | 1 " 11 | 1 to 190 | 1 to 12 | 8 " |
| Kentucky, | 1 " 178 | 1 " 9 | 1 " 106 | 1 " 6 | 1 to 142 | 1 to 7 | 14 " |
| Louisiana, | 1 " 228 | 1 " 13 | 1 " 97 | 1 " 10 | 1 to 162 | 1 to 12 | 8 " |
| Maine, | 1 " 68 | 1 " 4 | 1 " 42 | 1 " 4 | 1 to 55 | 1 to 4 | 25 " |
| Maryland, | 1 " 114 | 1 " 6 | 1 " 77 | 1 " 6 | 1 to 95 | 1 to 6 | 16 " |
| Massachusetts, | 1 " 72 | 1 " 4 | 1 " 49 | 1 " 5 | 1 to 60 | 1 to 4 | 25 " |
| Michigan, | 1 " 118 | 1 " 6 | 1 " 77 | 1 " 6 | 1 to 97 | 1 to 6 | 16 " |
| Minnesota, | 1 " 144 | 1 " 6 | 1 " 101 | 1 " 8 | 1 to 122 | 1 to 7 | 14 " |
| Mississippi, | 1 " 200 | 1 " 22 | 1 " 148 | 1 " 13 | 1 to 174 | 1 to 17 | 6 " |
| Missouri, | 1 " 220 | 1 " 13 | 1 " 161 | 1 " 10 | 1 to 190 | 1 to 11 | 9 " |
| Montana, | | | | 1 " 11 | 1 to 183 | 1 to 11 | 9 " |
| Nebraska, | 1 " 117 | 1 " 12 | 1 " 250 | 1 " 11 | 1 to 183 | 1 to 11 | 9 " |
| New Hampshire, | 1 " 43 | 1 " 4 | 1 " 36 | 1 " 5 | 1 to 39 | 1 to 4 | 25 " |
| New Jersey, | 1 " 100 | 1 " 5 | 1 " 75 | 1 " 6 | 1 to 87 | 1 to 5 | 20 " |
| New Mexico, | 1 " 326 | 1 " 38 | 1 " 69 | 1 " 26 | 1 to 197 | 1 to 32 | 3 " |
| New York, | 1 " 86 | 1 " 5 | 1 " 59 | 1 " 6 | 1 to 72 | 1 to 5 | 20 " |
| North Carolina, | 1 " 116 | 1 " 16 | 1 " 92 | 1 " 9 | 1 to 104 | 1 to 12 | 8 " |
| Ohio, | 1 " 104 | 1 " 7 | 1 " 70 | 1 " 6 | 1 to 87 | 1 to 6 | 16 " |
| Oregon, | 1 " 150 | 1 " 10 | | 1 " 6 | 1 to 150 | 1 to 8 | 12 " |
| Pennsylvania, | 1 " 95 | 1 " 6 | 1 " 69 | 1 " 7 | 1 to 82 | 1 to 6 | 16 " |
| Rhode Island, | 1 " 55 | 1 " 4 | 1 " 47 | 1 " 5 | 1 to 51 | 1 to 4 | 25 " |
| South Carolina, | 1 " 152 | 1 " 25 | 1 " 121 | 1 " 11 | 1 to 136 | 1 to 18 | 5 " |
| Tennessee, | 1 " 185 | 1 " 10 | 1 " 97 | 1 " 6 | 1 to 141 | 1 to 8 | 12 " |
| Texas, | 1 " 213 | 1 " 22 | 1 " 174 | 1 " 16 | 1 to 193 | 1 to 18 | 5 " |
| Utah, | | 1 " 18 | | 1 " 14 | | 1 to 16 | 6 " |
| Vermont, | 1 " 40 | 1 " 4 | 1 " 36 | 1 " 5 | 1 to 38 | 1 to 4 | 25 " |
| Virginia, | 1 " 133 | 1 " 10 | 1 " 87 | 1 " 7 | 1 to 110 | 1 to 8 | 12 " |
| Washington Territory. | | 1 " 6 | 1 " 45 | 1 " 6 | 1 to 45 | 1 to 6 | 16 " |
| West Virginia | | | 1 " 100 | 1 " 6 | 1 to 100 | 1 to 6 | 16 " |
| Wisconsin, | 1 " 129 | 1 " 7 | 1 " 60 | 1 " 8 | 1 to 94 | 1 to 7 | 14 " |

